# Tax inflicts DNA damage through activation of Nitric Oxide production

**DOI:** 10.1186/1742-4690-11-S1-P107

**Published:** 2014-01-07

**Authors:** Hicham H Baydoun, Lee Ratner

**Affiliations:** 1 Department of Medicine, Division of Molecular Oncology, Washington University in St. Louis, USA

## 

Adult T-cell Leukemia-Lymphoma (ATLL) is an aggressive and fatal malignancy of CD4+ T-lymphocytes associated with HTLV-1 infection, and an effective treatment is not yet available. The molecular mechanism underlying ATLL has not been fully elucidated. However, accumulation of genomic instability is believed to be a driving force for leukemogensis. How genomic instability accumulates in HTLV-1 infected cells is currently under intensive investigation. Recently, we found that the HTLV-1 viral oncoprotein, Tax, which is implicated in the chronic inflammatory response, induces DNA Double Strand Breaks (DDSB). Tax is known to activate the key T-cell inflammatory transcription factors, NF-kB, and this activation is critical for the leukemogenic process associated with HTLV-1 infection. Of note, we found that inducible nitric oxide synthase (iNOS), the enzyme that catalyzes the production of nitric oxide (NO) is highly expressed in HTLV-1 and Tax expressing cells. Interestingly, we show that the expression of iNOS is Tax-dependent and specifically requires the classical NF-kB pathway. In addition, IRF-1, the interferon regulatory factor that collaborates with NF-kB transcription factors to activate iNOS expression was also found activated by the JAK/STAT pathway. Our results show a correlation between the number of DDSB and the production of NO in tumors isolated from Tax transgenic mice. We also observed a dramatic reduction of DDSB when NO production was inhibited. Determination of the impact of NO on tumors in an ATLL mouse model will open a new area in the development of alternative strategies for the treatment/prevention of ATLL.

